# An Acoustic Sensor System to Measure Aeolian Ripple Morphology and Migration Rates

**DOI:** 10.3390/s24206555

**Published:** 2024-10-11

**Authors:** Pei Zhang, Jinsu Bae, Eric J. R. Parteli, Diane Sherman, Douglas J. Sherman

**Affiliations:** 1 USDA-ARS Jornada Experimental Range, Las Cruces, NM 88003, USA; peizhang@nmsu.edu; 2 Department of Geography, The University of Alabama, Tuscaloosa, AL 35487, USA; jbae12@crimson.ua.edu; 3 Faculty of Physics, University of Duisburg-Essen, 47057 Duisburg, Germany; eric.parteli@uni-due.de; 4 Independent Researcher, Tuscaloosa, AL 35406, USA; dianelynnbarron@gmail.com

**Keywords:** acoustic distance sensor, aeolian bedforms, signal processing, field method, data protocol

## Abstract

Acoustic distance sensors have a long history of use to detect subaqueous bedforms. There have been few comparable applications for aeolian bedforms such as ripples. To address this, we developed a simple and reliable apparatus comprising a pair of distance sensors, a bracket upon which they are mounted, and a base upon which the bracket can slide. Our system relies on two Senix Corporation (Hinesburg, VT, USA), ToughSonic^®^ model 14-TSPC-30S1-232 acoustic distance sensors: one to measure surface elevation changes (in this case, ripple morphology) and a second to measure horizontal location. The ToughSonic^®^ vertical resolution was 0.22 mm and the horizontal scan distance was about 0.60 m with a locational accuracy of 0.22 mm. The measurement rate was 20 Hz, but we over-sampled at 1 KHz. Signal processing involves converting volts to meters, detrending the data, and removing noise. Analysis produces ripple morphologies and migration rates that conform with independent measurements. The advantages of this system relative to terrestrial laser scanning or structure from motion are described.

## 1. Introduction

**Figure 1:**
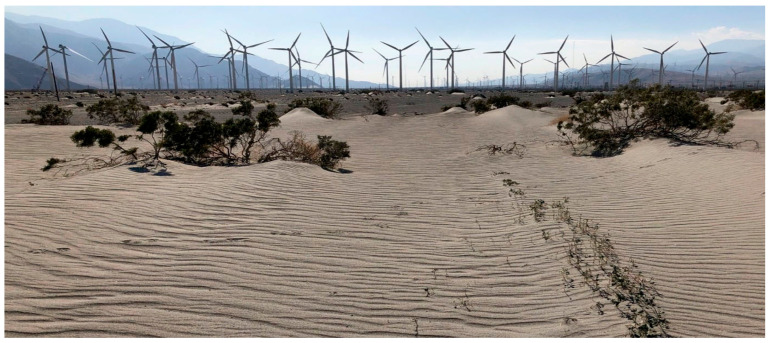
Multiscale aeolian ripples developed in an inter-vegetation saltation street near Palm Springs, CA, Amtrak station.

Aeolian ripples are a fine-scale geomorphological response to wind forcing over a deformable sand bed. They are common bedforms on Earth [1,2,3,4,5,6,7,8] and on extraterrestrial surfaces [9,10,11,12], and the regularity of the patterned surfaces they cause (Figure 1) has made them a focus of scientific attention for more than a century [1,13,14]. Ripple form is a morphological signature of wind speed and direction, responding to wind changes with adjustments to spacing, height, orientation, and migration rate. In accretionary environments, the stratigraphic signatures of morphological characteristics have the potential to be preserved in the rock record [9,15,16,17,18,19] and to be used in attempts at paleoenvironmental reconstructions [20,21]. Ripple migration rates are of interest because they are related to, and potential surrogates for, wind forcing, and the associated mass flux is one measure of creep transport [6].

Ripples form in many arid, fluvial, coastal, and extraterrestrial environments where there is a surface with unconsolidated or poorly consolidated sands and wind speeds sufficient to entrain those sands. The ripple-related literature from fluvial and coastal studies is extensive and detailed with regard to formation [22,23], modification [24,25], and migration [26,27]. These studies are often couched in the context of the flow regime concept [28,29]. Comprehensive studies of the formation, morphology, and migration of aeolian ripples are much less common. In a recent review, for example [6], the authors found very few field studies that reported concurrent measurements of ripple spacing and height, and readers are referred to that paper for more details concerning previous measurements. Those studies that do exist employed a range of sophistications, from manual measurements using rulers or tapes to terrestrial laser scanning and structure from motion technologies. Research has shown that acoustic distance sensors provide accurate and reliable results under the harsh, near-surface conditions associated with active sediment transport. Acoustic distance sensors are insensitive to ambient light conditions and dust [30], produce results comparable to those from terrestrial laser scanning (R^2^ > 90%) [31], and have accuracies of about 0.1 mm in an aeolian saltation layer [32]. Such characteristics make these sensors well suited for measuring aeolian sand ripples. In this paper, we describe a robust, acoustic distance-sensor system to measure ripple morphology and migration rates. The construction and deployment of the system and subsequent signal processing are detailed herein.

## 2. Previous Methods

Manual methods have dominated previous work to describe and quantify ripple morphology and migration in several fluid environments. Much of the fluvial literature is based on flume studies where ripples and their movement can be measured through transparent sidewalls or when flow through the flume has ceased [33,34]. In coastal systems, bedform morphology has often been studied by reconstructions based on cores or by direct observation by divers [35,36,37,38]. In aeolian systems, direct observation and measurement with rulers, for example, have been a standard, often supplemented with photography [3,6,39,40,41]. Repeat topography and digital terrain models have been used to measure ripple morphologies and, in some cases, migration rates on Mars, for example [42,43,44].

Technological advances have led to more sophisticated methods of ripple study, complementing or replacing the traditional approaches [45]. The development of high-resolution, subaqueous acoustic sensors is an early example of such advances. Side-scan sonar, for example, has long been used to detect and characterize ripple and other bedform morphology based on the return intervals of pulsed acoustic signals reflected from bed surfaces. Early applications were able to measure bedforms with lengths exceeding about 0.5 m [46,47]. Improvements in resolution soon allowed measurement at the vortex-ripple scale, ~ 0.1 m, and this became an important tool for environmental interpretation [48,49]. A further innovation that allows for the measurement of ripple morphology and migration is the installation of rotating side-scan sonar systems on a fixed surface mount ration, facilitating repeat bedform scans. This represents the subaqueous, acoustic analog to subaerial and subaquatic terrestrial laser scanning [50,51,52,53,54,55].

Greater advances in measuring bedform morphology and migration have been accomplished through the application of remote sensing techniques to derive the relevant data from satellite images, terrestrial laser scanning (TLS), structure from motion photogrammetry (SfM), and particle tracking/imaging velocimetry (PTV/PIV). Satellite images have been the only reliable means of measuring bedforms on extraterrestrial surfaces, even though the image resolutions are relatively coarse [9,10]. Recently, curiosity rovers have provided high-resolution photographs allowing for the observation of the detailed morphology of Martian surface ripples, which are composed of two distinctive forms with large (bigger than 0.8 m) and small (smaller than 0.2 m) wavelengths [9,11,56,57].

The TLS, SfM, and PTV/PIV approaches are capable of detailed spatial and temporal resolutions, although they are currently limited to terrestrial applications. Terrestrial laser scanning involves machine-driven rotation of a tripod-mounted LiDAR system that emits a pulsed laser signal. Return intervals of reflections from a surface are recorded and used to produce three-dimensional point clouds that can be converted to digital elevation models (DEMs). TLS has been used to measure bedform characteristics in shallow aquatic environments [58,59,60,61,62] and aeolian environments. For example, Nield et al. [63] used a 5 mm resolution TLS to image a rippled sand surface with a grid resolution of 2 cm. Squirrell [64] used a 1.4 mm resolution TLS to assess the relative influences of bedform geometry, scan incident angle, scanning time, wind speeds, and orientation of the scanner on the spatial resolution of the consequent measurements. Trimble et al. [65] set up a configuration of TLS with an 8 mm accuracy, a sonic anemometer, a cup anemometer, and traps to detect ripple geometry, as well as migration rates relating to sand flux and wind velocity. Li et al. [66] used a 1.6 mm resolution TLS to measure surface ripple geometry where the height of the ripples was smaller than 15 cm, allowing for the collection of longitudinal profile data. Individual scans with state-of-the-art TLS methods can produce millimeter-scale measurements of ripple heights and lengths. Repeat scans at intervals of 100 s magnitude allow for accurate, simultaneous measurements of the migration rates of multiple ripples and the derivation of migration statistics therefrom. We found no TLS-based examples of sand ripple morphology or migration studies reported in the literature.

Structure from motion (SfM) techniques involve obtaining overlapping digital images (typically photographs) of an object by moving the sensor around that object. The geo-rectified images can be photogrammetrically combined to develop a three-dimensional point cloud from which one can derive a DEM. Mobile LiDAR systems (as opposed to stationary TLS systems) are also used in SfM studies. The DEM resolution depends on the nature of the sensor used and the distances from which the images are acquired, and images may be obtained using hand-held or drone (UAV)-mounted sensors. SfM has been applied to study millimeter- to centimeter-scale hydrodynamic ripples [67,68,69,70] and aeolian ripples, although there are relatively few of the latter with very little concerning specific ripple geometry. Luo et al. [71] used SfM to identify ripple texture in a large blowout but did not measure specific ripple geometry. Similarly, Shumack et al. [72] used the method to identify rippled surfaces as related to vegetation geometry. Several applications of SfM have been used to describe terrestrial and Martian megaripples [66,73]. Coarse grain ripples on Earth and Mars have also been analyzed using SfM approaches [41,74,75]. The emphasis on megaripples and coarse grain ripples is a consequence of resolution challenges. We found no SfM-based examples of sand ripple morphology or migration studies reported in the literature. While the PTV/PIV technique has mainly been used to elucidate interactions of fluid–sediment flow fields and evolution of sand ripple morphology [76,77], ripple geometry could be derived from such measurements. Table 1 summarizes examples of previous studies that involve measuring ripple morphology and migration, which are categorized according to measurement methods, and describes the reported measurement resolutions. The advantages and disadvantages of the approaches are summarized in the discussion.

## 3. Acoustic Distance Sensors and the Ripple Rack System

The conceptual foundation of the ripple rack system described herein lies with the acoustic ripple profiler described by Dingler et al. [91]. This device comprised a 4.5 mHz transducer capable of 1 mm vertical resolution, mounted on a movable bracket on a 2 m aluminum frame. The transducer was displaced along the frame by a diver-operated drive mechanism that was linked to a potentiometer to link horizontal and vertical measurements and thus allow for the reproduction of ripple profiles. A more sophisticated version of the ripple profiler was the high-resolution remote tracking sonar system developed by Greenwood et al. [92,93]. This profiler was controlled via a shore-based computer that was connected to the system with cables. The sonar provided a vertical resolution of a few mm and was moved along a 5 m track with horizontal positions measured with a potentiometer.

A previous application of acoustic distance sensing to aeolian ripples involved two sensors pointing toward a rippled surface (described in Sherman et al. [6]). That use lacked direct measurement of ripple wavelengths and relied on cross-correlation analysis to estimate those lengths and, thus, migration rates as well. Such issues are overcome by the ripple rack system that we devised for aeolian applications. It comprises a set of acoustic distance sensors to measure horizontal and vertical distances and is attached to a sliding bracket on a mounting rack, with associated power, data acquisition, and recording accessories. The acoustic distance sensors we employed were sets of Senix ToughSonic^®^ model 14-TSPC-30S1-232. Each sensor was hardwired to a power supply and laptop computer system that controlled data acquisition and recording. Our ToughSonic^®^ model requires an input voltage in the range of 10 to 30 VDC, and we used an output signal range of 0 to 10 VDC. Senix-provided software (SenixVIEW Version 3.6.300) was used to set a distance range that required the sensor to be no closer to a surface than 0.10 m and no further than 1.00 m. We used the sensor default measurement rate of 50 msec (20 Hz), but logged the data at 1 kHz for redundancy. This sample rate, when coupled with other measurements (described below), would produce more than 30 samples per 100 mm long ripple. Data storage capacity was not an issue for us, but might otherwise have constrained the oversampling. The distance resolution (vertical and horizontal) with our ToughSonic^®^ settings was 0.22 mm, based on the 900 mm between the maximum and minimum sensing ranges and the 4099 signal increments recognized by the sensor when in voltage output mode.

**Figure 2:**
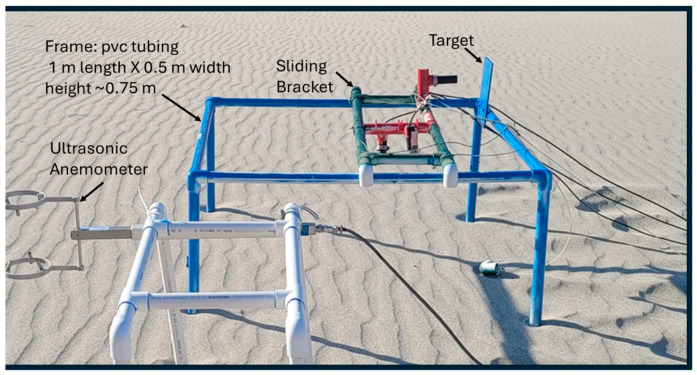
The ripple rack mount and the ToughSonic^®^ bracket, paired with a 3D, ultrasonic anemometer (SATI/3A-130201). The cables lead to the power supply, data acquisition, and recording hardware.

**Figure 3:**
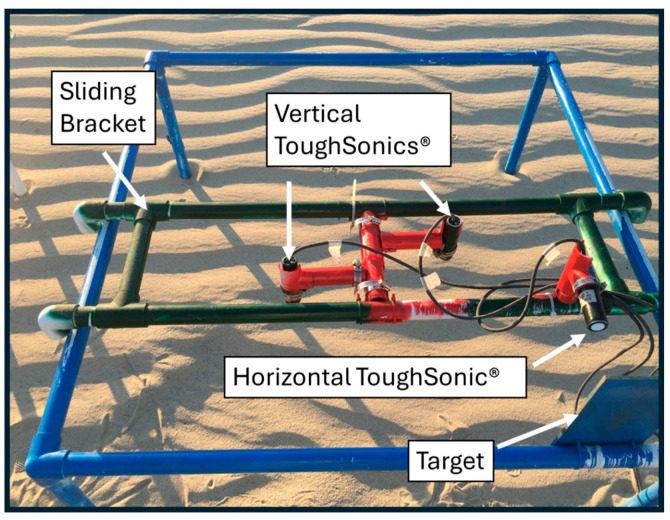
Details of the sliding instrument bracket (green paint) with three ToughSonics^®^: one mounted horizontally and two mounted vertically.

The mounting rack was designed to be a stable platform to support a sliding bracket to which the ToughSonic^®^ is attached (Figure 2 and Figure 3). Our version was constructed with PVC tubing and fittings, although other materials such as wood or metal would also work and would potentially provide a more stationary frame. The version described herein was designed for deployment at Windy Point (33°53′45.28″ N; 116°38′46.06″ W: described in Baas and Sherman [94]) in the Santa Rosa and San Jacinto Mountains National Monument, administered by the US Bureau of Land Management. Vehicle access to the site was prohibited, and the instruments needed to be installed and removed daily. Therefore, we used a lightweight (PVC-based), portable version of the ripple rack system that could be easily carried and quickly assembled and installed. In this version, the ToughSonic^®^ bracket and rack legs were detachable from the main body. The legs were dry-fit to PVC couplings that had been cemented to the body (blue paint in Figure 2). Slight variability in leg lengths resulted from the imprecise nature of the dry-fit. The absolute length is not critical, as the resulting height of the bracket above the surface depends on the depth to which the legs are driven. We aimed to have the sensors approximately 0.50 to 0.60 m from the surface.

The sliding bracket used here was also constructed from PVC pipes, and 90 degree elbows were attached to both sides of the bracket in order to guide the bracket as it was pushed and pulled along the frame. Each of the ToughSonic^®^ sensors was mounted onto the sliding bracket with 1 ½ inch PVC “T” fittings, which were cut and heated to mold to fit the sensors to simplify their attachment to the bracket. The horizontal sensor was aimed at a stationary target attached to the back of the frame. The two vertical sensors were aimed at the surface to measure the elevation changes.

The data were acquired in two modes. The first was a stationary mode where the bracket was motionless, and the vertical sensors monitored the elevation changes caused by migrating ripples. The second was the sliding mode, where the bracket was manually pushed to its stops near the target and then pulled back to the opposite stop. Transit times were approximately ten seconds each way to minimize effects of ripple movement. Unless the transit time was long relative to ripple migration rates, the consequent measurement error would be small. Lag effects were nil at the beginning of the slide and maximal at the end of the slide. The horizontal sensor tracked the distance traversed and, hence, the locations of the vertical sensors.

## 4. Signal Processing

**Figure 4:**
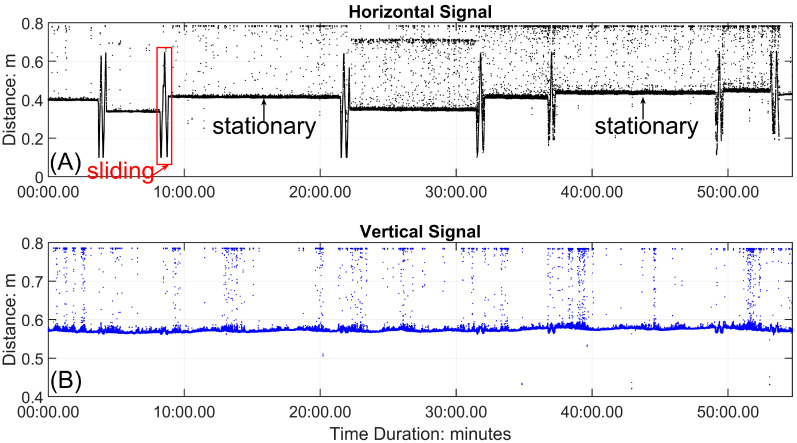
ToughSonic^®^ time series from ripple rack measurements made on 10 May 2022. Data are calibrated but otherwise unprocessed. (**A**) is the horizontal signal with periods when the instrument bracket is sliding to measure ripple heights and lengths or when it is stationary to measure migration rates. (**B**) is the vertical signal. When the bracket slides, these data match ripple heights with lengths obtained by the horizontal sensor. When the bracket is stationary, the vertical measurements document the elevation changes associated with ripples migrating beneath the sensor.

The panels in Figure 4 illustrate unfiltered, calibrated time series from a 55-min-long dataset that began at 18:10:22 (PDT) on 10 May 2022, from instruments installed at the top of a barchan in the dune field near the Salton Sea, California, USA (described by Pelletier [95], for example). Figure 4A depicts the time series of the signal from the horizontally oriented ToughSonic^®^ sensor. Distances are those from the sensor to the target at the upwind end of the ripple rack that was used to measure the changing position of the vertically oriented ToughSonic^®^ as it traversed. When the sensor was stationary, the signal was steady at a constant distance, as labeled in the figure. Note that the offsets in the stationary signals were caused when the bracket was not returned to its exact starting position. These offsets can be corrected digitally by aligning the crests in the direction of migration, or avoided by marking precise start and stop locations for the slide. When the bracket was moved quickly forward and back (twice each time—labeled as sliding in the figure) along the rack, the signal varied over a range of about 0.6 m. Figure 4B depicts the time series of the signal from the vertically oriented ToughSonic^®^. Both sets of signals included substantial noise arising from electronic noise in the system (random distances), acoustic beam interference from saltating grains (shorter distances), minor vibrations in the frame caused by wind gusts, and, most commonly, echo noise caused by multiple reflections of the same acoustic beam (longer distances). The magnitude of noise included in Figure 4B, without processing, masks the surface signal. Two stages of noise removal are typically required. In the first stage, data points that are well outside the range of the signal (greater than 0.60 m in Figure 4A, for example) are identified and deleted, with the consequent gap filled via linear interpolation from adjoining points. The second stage involves removing noise at the scale of the signal—a point of the same magnitude as a ripple crest elevation but located over a trough, for example. Such points can be identified and deleted using moving averages and replaced using linear interpolation with the procedure detailed below, which is illustrated with examples.

**Figure 5:**
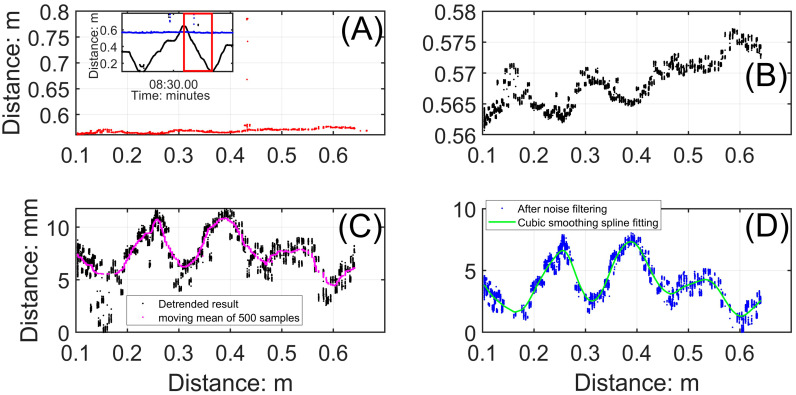
Processing the “sliding” signal to obtain estimates of ripple spacing and height. (**A**) Calibrated but unprocessed signal, indicating its place in the complete time series. (**B**) The time series after the first stage of noise removal. (**C**) The time series after inversion, detrending, and fitting a moving average. (**D**) The signal (green line) after a second smoothing.

The panels in Figure 5 depict the steps to process parts of the time series from Figure 4 to obtain representations of ripple morphology. Figure 5A (inserted subplot) includes the measurements obtained from one unidirectional slide of the horizontal ToughSonic^®^; in this case, it is from the right of the red box indicated in Figure 4A, at about 8 min in the time series. After plotting the horizontal distance signal on the *x*-axis and the vertical distance signal on the *y*-axis (Figure 5A), the noise in these data obscured the surface morphology. Because they were so different from the signal, those points were simple to identify as outliers and then to remove. Figure 5B depicts the signal after the obvious outliers had been removed, and, as a consequence, the surface signal is obvious. Figure 5C depicts the next two steps in the signal processing. First, the larger vertical distances registered by the ToughSonic^®^ indicate lower elevations of the surface because it is farther from the sensor. Therefore, the time series was inverted by subtracting individual distance (elevation) values from a maximal distance, which was 0.58 m in this example. All elevations were relative to one another but could be converted to absolute elevations if tied to a local reference. Second, there was an obvious trend in surface elevations in Figure 5B because the ripple rack was not installed parallel to the surface. This was corrected by detrending the data using linear regression and plotting the residuals. If noise was still apparent in the data after vertical signal inversion and detrending, further filtering became desirable; in this case, this occurred especially at about 0.15 m distance.

Substantial noise remained after the first three steps, as seen in Figure 5C. The signal was processed by fitting a 500-point moving average (the magenta line in Figure 5C). The size of the moving average was based on the 1 KHz sample rate we used. Equation (1) was used to identify substantial departures from the average, and, in this case, that procedure was repeated to obtain the “clean” signal depicted in Figure 5D.

(1) $$\left|SV_{i}-\bar{SV_{i}}\right|>\bar{\left|SV_{l}-\bar{SV_{l}}\right|}+\sigma \left(\left|SV_{i}-\bar{SV_{l}}\right|\right)$$

where $SV_{i}$ is a vertical sample; the overbar indicates mean values; $\bar{SV_{l}}$ is the corresponding 500-point moving average with sample i in the center, as calculated from the ‘movmean’ function in MATLAB R2023b; and σ represents the standard deviation. Spurious samples are made obvious because of their degree of separation from the moving average. Finally, a cubic spline curve is fit to the remaining data (the green line in Figure 5D). The curve is used to calculate mean ripple spacing (trough to trough) and height (crest to trough): 145.8 mm and 4.6 mm, respectively.

**Figure 6:**
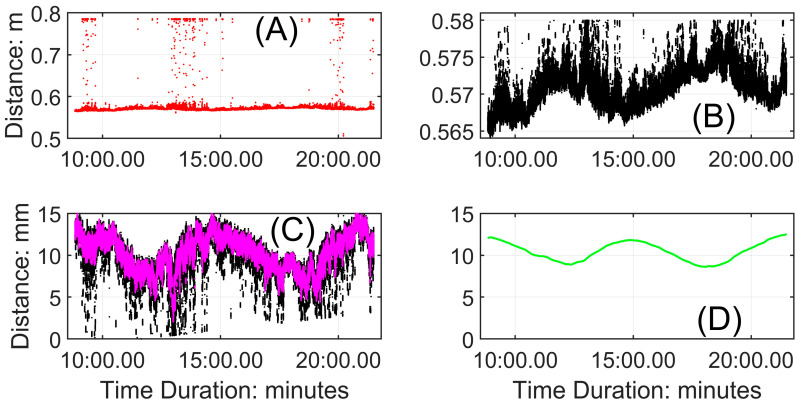
Processing the “stationary” signal to obtain estimates of ripple migration rates. (**A**) Calibrated but unprocessed signal, indicating its place in the complete time series. (**B**) The time series after the first stage of noise removal. (**C**) The time series after inversion, detrending, and fitting a moving average. (**D**) The signal (green line) after a second smoothing. The ripple migration rate is calculated based on the time and distance separating the two troughs.

**Figure 7:**
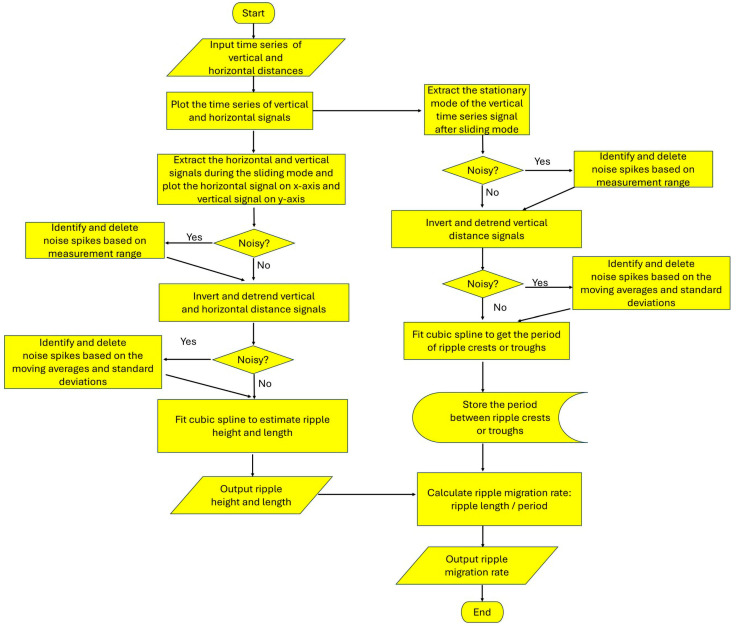
Flow diagram summarizing the data-processing procedure to produce estimated ripple height, length, and migration rates. Generic steps are depicted rather than the specifics detailed in the text.

A similar procedure is used to process the signal from the stationary mode of the vertically oriented ToughSonic^®^. The example here is from the stationary period indicated in Figure 4B, from about 10 to 22 min. Figure 6A is the raw extracted signal, which contains substantial noise. Figure 6B is the preliminary filtered result based on the sensor’s maximum distance to the surface (0.58 m). Figure 6C is the signal (represented in magenta) after inverting and detrending the vertical distances (as described above) and filtering out noise, achieved by applying the smoothing and noise removal process twice using Equation (1), which is also described above. Figure 6D depicts the processed signal after fitting a cubic spline. Based on the time interval (362 s) between two troughs passing beneath the sensor and the corresponding distance of 128 mm, the ripple migration rate is determined to be 0.35 mm/s. The values we obtained from these examples (ripple heights, lengths, and migration rates) conform closely to our independent measurements and values found in the literature [6]. The error associated with the 10-s bracket sliding time, relative to the ripple migration rate, is of the order of 1%. Under the sand size and wind conditions (with an average shear velocity to threshold shear velocity ratio of 2.2) during the sample run used here, ripple heights ranged between 4 and 6 mm, and wavelengths ranged between 100 and 150 mm. Comparable ripple migration rates for similar conditions can be found in Table S1 in Sherman et al. [6]. The data processing procedure is summarized in Figure 7.

## 5. Discussion

The ripple rack system is a reliable and relatively simple means to obtain high-quality measurements of ripple morphology and migration rates. The system is logistically efficient in that setup and preparation times are minimal and data-efficient because relatively few extraneous data points are produced. Consequent data processing requirements are minimal, in sharp contrast to the processing associated with the point cloud and DEM productions required with TLS or SfM studies. The rack, bracket, and sensors are individually and collectively robust, capable of continuous measurement at night and under typical aeolian conditions when blowing sand and dust are present [30], and the sensor can be used in marine environments [88]. Occasional cleaning of the ToughSonic^®^ transponder head might be necessary if it becomes coated with salt or dust. The paired distance sensors provide positional accuracies at sub-millimeter scales: 0.22 mm in our application and 0.1 mm in others [32,88].

The ripple rack we describe can be assembled for about USD 1500 with two ToughSonic^®^ acoustic sensors, or for less than USD 2000 with three, excluding power supply and data acquisition systems. These costs are small relative to the cost of purchasing a TLS system, which ranges over a decade of magnitude, from about USD 12,000 to more than USD 150,000 as of this date. The wide range of costs mainly reflects differences in scanner range and distance resolution. TLS systems for accurate and precise ripple scanning will be toward the more expensive end of the range because the small heights of the features suggest that precisions of 1 mm or less are desirable. This resolution was not attained by the TLS systems we have reviewed in the literature. Typical sand ripple heights generally range from 5 to 10 mm; therefore, a TLS resolution of 1 mm would be associated with measurement errors of 10 to 20% based on manufacturer’s claims [96,97]. However, these claims should be treated with some skepticism [98].

Technologies to obtain suitable SfM data for ripple measurement are available at much lower costs than TLS. These data can be obtained with a handheld or drone-mounted smartphone, for example, with hardware costs as little as, perhaps, USD 2500. The use of some drone models to obtain SfM data to measure ripple migration rates may be impractical because of the difficulties associated with wind speeds common during sand transport events. SfM measurement also requires the placement and assessment of stable ground control points or targets [99,100], which is not necessary with the ripple rack system.

Terrestrial laser scanning and structure from motion methods generate very large data sets that often require extensive processing and, in some cases, careful registration against ground control points. Single data sets from either technology may require several hours to many days to process, depending on the file sizes and computation resources available. As outlined above, ripple rack data are relatively simple to analyze, with individual runs requiring 10–15 min with packaged programs. Noise removal is the major challenge and may require a trial-and-error approach to discover an optimal moving average for signal smoothing (similar to the process described by Martin and Jerolmack [45]). As a cautionary note, it is important to recognize that smoothing reduces the heights of ripple crests and raises the heights of the troughs. Excessive smoothing, therefore, will indicate ripple relief less than is actual. 

The concept underlying the ripple rack allows for substantial flexibility in alternative designs. A larger frame made of PVC tubing would be subject to shaking under strong wind conditions; thus, rigid materials would be an obvious substitute, especially where portability is not a consideration. The greater length would allow a larger number of ripples to be sampled during each pass of the sliding bracket. Greater width would allow for the use of additional vertical sensors so that several ripple profiles could be measured simultaneously and a 3D rendition of the morphology could be produced. Additional care should be taken for the locations of vertical sensors to avoid signal distortion by the presence of the legs of the mount. Scouring or distortion of ripples occurs within approximately 0.1 m of the bases of the poles, indicating that their horizontal spacing should be at least 0.3 m. Installing one ToughSonic^®^ on the upwind side of the mount allows for the continuous, vertical monitoring of ripple migration, independent of the sliding bracket.

A ripple rack could also be used in combination with surface scanning technologies such as TLS or structure from motion data acquired from a drone or smartphone. Some of the data-processing challenges associated with the frequent scanning necessary to measure ripple migration are ameliorated by the less frequent scanning necessary to monitor morphology only. Morphology-only scans could occur at intervals of 15 to 60 min under conditions of relatively constant wind speed and direction. In some cases, the high cost of TLS systems or restrictions on drone usage may make such parings impractical.

With the portable system we used, it was easy to align with regard to ripple orientation. With larger, heavier systems, this might be problematic, so a digital correction for ripple spacing would be appropriate. The long axis of the ripple rack should be aligned perpendicular to the trend of the ripple crests. During a longer run, however, the wind direction and, therefore, ripple migration can significantly depart from the original orientation. Departures from the perpendicular can be measured with a protractor or compass, and the relative angle can be used to adjust the distances used for ripple spacing and migration rates. This may only be necessary when the angle becomes less than about 75°, as that offset would cause an error of less than 4%, which would be trivial in most applications (sine 75°~0.966).

Each of the fundamental approaches to measuring ripple morphology and migration rates has advantages and disadvantages. Manual methods are relatively simple, requiring no technology, but needing dedicated labor. Data analysis is simple. Ripple spacing is typically estimated by measuring the distance between 11 ripple crests and dividing by 10. This produces an average spacing to reduce the chances of measuring an individual atypical ripple. Successive measurement of crest movement through time, relative to a known point, allows for migration rate estimation. These measurements can be accomplished with accuracies better than 10 mm if conducted with care. Ripple heights are much more difficult to measure because of their small heights, ripple-to-ripple variability, and the difficulty in measuring without disturbing the sand surface. Optical sensors and approaches such as TLS and SfM, as used in field applications, have the advantage of being relatively simple to deploy and can provide a large coverage area suitable for deriving morphology statistics, for example. It appears that, at present, both technologies lack the vertical (in particular) accuracy necessary for the characterization of small ripple heights, and this disadvantage makes them unsuitable for ripple measurements, as described herein.

## 6. Conclusions

The acoustic sensor-based ripple rack system is an efficient, robust, and accurate means to measure aeolian ripple morphologies and migration rates. The system concept allows for numerous modifications to the version described herein, including larger frames made with stiffer materials, a greater number of vertically oriented sliding sensors, and one or more fixed vertical sensors. Increasing the number of sensors provides a basis for the appraisal of three-dimensional aspects of ripple sets.

Relative to terrestrial laser scanning or structure from motion approaches, the ripple rack system either is inexpensive, produces data sets that are easy to process, or both. Our system used the Senix Tough Sonic^®^ distance sensors, but any similar acoustic device would be suitable. The ripple rack system is designed to be simple to construct, deploy, and operate. It produces accurate and reliable representations of ripple morphology and migration rates. Given the increasing attention to aeolian ripples as sensitive geomorphological responses to fluid forcing, it is somewhat surprising that the development of a system such as this has not occurred earlier.

Aeolian ripples are minor landforms that can be important indicators of transitory environmental conditions. Morphological signatures can represent different wind speeds and directions. Migration rates can represent supra-threshold wind speeds, can be surrogates for aeolian creep transport, and are clear indicators of an active aeolian system. Toward these and other ends, accurate, precise, and reliable measurements are necessary. The ripple rack system described in this paper is the only non-manual approach that can accomplish those necessities. Future applications to closely link wind conditions with dependable ripple measurements will further our understanding of aeolian sand transport systems, improve stratigraphically based paleoenvironmental reconstruction, and provide baseline conditions against which to detect climate change.

## Figures and Tables

**Table 1:** Examples of published ripple studies based on research approaches and methods. Few studies include both ripple morphology and migration rates.

Approach	Environments	Reference	Resolution	Morphology	Migration Rates
**Manual**	Aeolian (Lab)	Bagnold [78]	N/A	Y	N
	Aeolian (Field)	Sharp [3]	N/A	Y	Y
	Aeolian (Lab)	Seppälä and Lindé [79]	N/A	Y	Y
	Aeolian (Field)	Han et al. [41]	1 cm	Y	N
	Coastal (Field)	Davidson-Arnott and Greenwood [35]	N/A	Y	N
	Coastal (Field)	Short [36]	N/A	Y	N
	Coastal (Lab)	Sarkar et al. [23]	N/A	Y	Y
	Fluvial (Lab)	Gilbert [26]	N/A	Y	Y
	Fluvial (Lab)	Raudkivi [22]	N/A	Y	Y
	Fluvial (Lab)	Allen [80]	N/A	Y	N
	Fluvial (Lab)	Costello and Southard [24]	1 cm	Y	Y
**Optical**					
Photography	Aeolian (Lab)	Cheng et al. [40]	N/A	Y	Y
	Aeolian (Field)	Lorenz [81]	N/A	N	Y
	Aeolian (Field)	Sherman et al. [6]	1 mm	Y	Y
	Coastal (Lab)	Koller et al. [33]	N/A	Y	N
LiDAR	Aeolian (Lab & Field)	Andreotti et al. [82]	40 μm (Lab)	Y	Y
	Aeolian (Field)	Nield et al. [63]	5 mm	Y	N
	Aeolian (Field)	Squirrell [64]	1.4 mm	Y	Y
	Aeolian (Lab)	McKenna-Neuman and Bedard [83]	2 mm hor., 10 μm ver.	Y	Y
	Aeolian (Field)	Field and Pelletier [84]	2 mm	Y	N
	Aeolian (Lab)	Wang et al. [85]	0.5 mm	Y	N
	Aeolian (Field)	Li et al. [66]	1.6 mm	Y	N
	Coastal (Lab)	Jin et al. [86]	2 mm hor.	Y	N
	Coastal (Lab)	Lee et al. [87]	3 mm	Y	Y
SfM	Aeolian (Field)	Qian et al. [75]	2.9 cm	Y	Y
	Coastal (Field)	Bertin et al. [69]	1.5 cm ver.	Y	N
	Fluvial (Field)	Li et al. [68]	0.5–1 mm	Y	N
**Acoustic**	Aeolian (Field)	Houser [32]	0.1 mm	Y	Y
	Coastal (Field)	Houser and Barrett [88]	0.1 mm	Y	Y
	Coastal (Field)	Flemming and Bartholomä [89]	N/A	Y	Y
	Coastal (Field)	Wengrove et al. [27]	2.5 cm at 1 m	Y	Y
	Coastal (Field)	van der Werf et al. [38]	1 cm	Y	Y
	Fluvial (Lab)	Martin and Jerolmack [45]	0.25 mm ver.	Y	Y
	Fluvial (Lab)	Bradley and Venditti [90]	N/A	Y	N
	**Aeolian (Field)**	**This Paper**	**0.22 mm**	**Y**	**Y**

## Data Availability

No new data were created for this methods paper. All relevant information is presented herein.
